# Conjugated Linoleic Acid-Mediated Connexin-43 Remodeling and Sudden Arrhythmic Death in Myocardial Infarction

**DOI:** 10.3390/ijms241311208

**Published:** 2023-07-07

**Authors:** Natia Qipshidze Kelm, Jane C. Solinger, Kellianne M. Piell, Marsha P. Cole

**Affiliations:** 1 Department of Biochemistry and Molecular Genetics, Louisville, KY 40202, USA; 2 Department of Physiology and Biophysics, School of Medicine, University of Louisville, Louisville, KY 40202, USA

**Keywords:** conjugated linoleic acid (cLA), connexin 43 (Cx43), NADPH oxidase (NOX), reactive oxygen species (ROS), myocardial infarction (MI)

## Abstract

Connexin 43 (Cx43) is expressed in the left and right ventricles and is primarily responsible for conducting physiological responses in microvasculature. Studies have demonstrated that NADPH oxidase (NOX) enzymes are essential in cardiac redox biology and are responsible for the generation of reactive oxygen species (ROS). NOX2 is linked to left ventricular remodeling following myocardial infarction (MI). It was hypothesized that conjugated linoleic acid (cLA) treatment increases NOX-2 levels in heart tissue and disrupts connexins between the myocytes in the ventricle. Data herein demonstrate that cLA treatment significantly decreases survival in a murine model of MI. The observance of cLA-induced ventricular tachyarrhythmia’s (VT) led to the subsequent investigation of the underlying mechanism in this MI model. Mice were treated with cLA for 12 h, 24 h, 48 h, or 72 h to determine possible time-dependent changes in NOX and Cx43 signaling pathways in isolated left ventricles (LV) extracted from cardiac tissue. The results suggest that ROS generation, through the stimulation of NOX2 in the LV, triggers a decrease in Cx43 levels, causing dysfunction of the gap junctions following treatment with cLA. This cascade of events may initiate VT and subsequent death during MI. Taken together, individuals at risk of MI should use caution regarding cLA consumption.

## 1. Introduction

Conjugated linoleic acid (cLA) refers to a group of positional and geometrical isomers of octadecadienoic acid, with two alternating double bonds. Rumen bacteria has the unique ability to convert linoleic acid into cLA via an enzymatic isomerase reaction, and therefore at least 28 possible isomers of linoleic acid are found in meat and dairy products [1]. The prominent cLA isomers that are commercially available as dietary supplements and in food products are mixtures of cis-9, trans-11-cLA and trans-10, cis-12-cL, with cis-9, trans-11 accounting for 72–94% of total cLA in foods. There is emerging evidence that individual cLA isomers may be responsible for specific biological or biochemical changes in the body. Most studies use isomeric mixtures, as there are 28 different geometric and positional isomers of cLA [1]. However, isomers cis-9, trans-11-cLA and trans-10, cis-12-cLA are the only two isomers to date that have been linked to specific biological effects. For example, both isomers have been shown to inhibit carcinogenesis. However, individually, cis-9, trans-11 is mainly responsible for anticarcinogenic effects, whereas the trans-10, cis-12 isomer reduces body fat and is commonly referred as the most effective isomer affecting blood lipids.

Numerous studies have reported positive effects of cLA consumption, including weight loss in obese individuals [2], modulation of immune function [3,4] anti-carcinogenic activity [5], protection against atherogenesis [6,7], reversal of atherosclerosis [8], and normalization of glucose and insulin homeostasis in pre-diabetic animal models [9,10]. Additionally, the Food and Drug Administration notably reported that cLA as being “generally regarded as safe” in 2008. However, we have since shown that cLA treatment lowers physiological nitric oxide (NO) levels and impairs heart function in aged mice [11]. Following these findings, we wanted to determine if survival was also affected as a result of impaired heart function.

Connexins are gap-junction transmembrane channels that mediate the cell-to-cell movement of small molecules and/or ions to maintain intercellular homeostasis [12]. Gap junctions contain transmembrane ion-channel proteins which are cylinders constructed from 6 copies of transmembrane proteins, otherwise known as connexins [12]. In the heart, connexins mediate ion flow through the cardiac myocytes, forming intercellular pathways that allow for the organization of electrical excitation responsible for heart muscle contraction [13,14].

Approximately twenty connexins have been identified in the mouse genome and twenty-one in the human genome, where orthologous are increasingly characterized in other vertebrates [15]. There are 3 main connexins expressed in the heart, including Connexin-43 (Cx-43), Connexin-40 (Cx-40), and Connexin-45 (Cx-45) [16]. Previous studies have shown that Cx-43 is highly expressed in both ventricles, where both atria equally express Cx-43 and Cx-40 [16,17]. Myocytes of the sinoatrial and atrioventricular nodes normally have small, isolated gap junctions composed of Cx-45 [16,18]. Since it is known that connexins are of essential importance during intercellular communication, changes in connexin expression and distribution are expected to have insightful impacts on cardiac conduction and the generation of arrhythmias. Previous studies have shown Cx-43 to be an arrhythmogenic junction in the heart [16,19]. Lerner et al. have shown that Connexin-43 (Cx43)-deficient mice were susceptible for ischemia-induced VT [20].

Excess amounts of reactive oxygen species (ROS) have been implicated in the genesis of arrhythmia by altering Cx-43 [21]. NADPH oxidase (NOX) enzymes have been implicated in cardiac redox biology and are responsible for the generation of ROS [22,23]. Of the NADPH oxidase homologs, NOX2 and NOX4 are most abundantly expressed in the myocardium [24]. Under physiological conditions, nonphagocytic NADPH oxidase enzymes generate lower levels of intracellular ROS that are necessary for cell signaling, adaptation and survival, unlike phagocytic NADPH oxidase, which releases a large “respiratory burst” of superoxide (O_2_^•^) for pathogen extermination [23]. The NOX family is composed of seven members (Nox1–Nox5, Duox1, and Duox2) that transfer electrons across biological membranes to generate ROS [24]. NOX2 has been linked to left ventricular remodeling after myocardial infarction [25].

In the present study, we wanted to further examine the effects and potential mechanisms of cLA treatment on MI. Our results indicate that cLA treatment significantly decreases survival in a murine model of myocardial ischemia (MI), causing life-threatening spontaneous ventricular tachycardia (VT) and sudden cardiac death (SCD). Time point treatment was used to further elucidate the possible mechanism whereby cLA induces life-threatening arrhythmias. Herein, we demonstrate that cLA significantly induces levels of NOX2 expression and ROS generation in myocytes, which results in the disruption of connexins between the myocytes in the ventricle, causing life-threatening arrhythmias and subsequent death in mice during MI.

## 2. Results

### 2.1. Electrocardiogram (ECG) Recordings Prior to MI Surgery Demonstrate VT following Treatment with cLA

**Figure 1:**
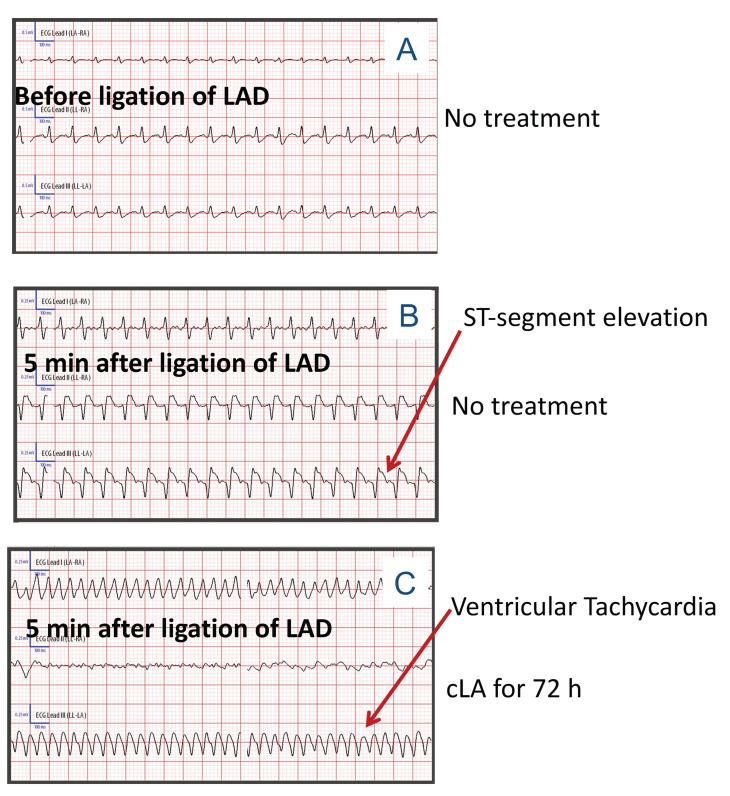
cLA treatment induces ventricular fibrillation following MI. ECG data from control and cLA-treated mice (**A**) before MI, (**B**) 5 min after MI in control mice, and (**C**) 5 min after MI in cLA-treated mice demonstrate changes in ST segment elevation following LAD. MI surgery in untreated mice (n = 15); MI surgery in cLA-treated mice (n = 35).

Structural and functional changes following MI are characterized by using non-invasive ECG to determine post-infarct cardiac remodeling. During the MI surgery, electrocardiogram (ECG) recordings in untreated mice detected normal sinus rhythms (Figure 1A). Following the ligation of LAD, mice without treatment developed ST-segment elevation, which is an earliest sign of MI (Figure 1B). Previous studies revealed that reduction of Cx-43 expression increases frequency of spontaneous ventricular ectopy and arrhythmogenesis compared to control mice [26]. Mice treated with cLA developed VT and died within 1–2 min following ligation of LAD (Figure 1C).

### 2.2. Treatment with cLA Does Not Affect Cardiac Function before MI

**Figure 2:**
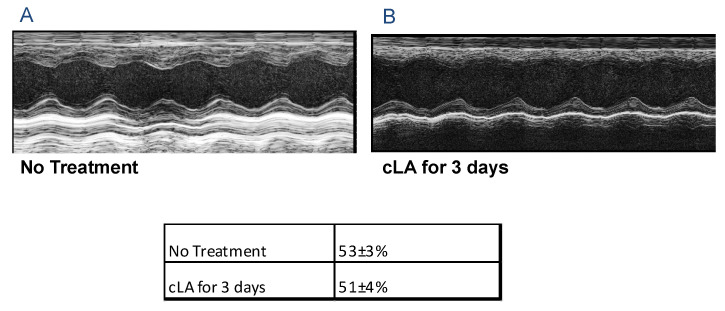
cLA treatment does not affect cardiac dysfunction after 72 h of treatment. Treatment with cLA does not affect cardiac function, FS in (**A**) control and (**B**) cLA-treated mice were 53 ± 3 and 51 ± 4, respectively. MI surgery in untreated mice (n = 15); MI surgery in cLA-treated mice (n = 35).

To establish baseline myocardial function, echocardiography was performed in 3-month-old mice (untreated). The analysis of left ventricular function, determined via percentage of fractional shortening (%FS), demonstrated that there were no significant changes in cardiac function between untreated and treated groups (Figure 2A,B). FS was normal in both control (53 ± 3%) and cLA-treated (51 ± 4%) groups (Figure 2 Table). These results indicate that cLA does not cause adverse myocardial changes in the cardiac function of normal, healthy mice. However, cLA treatment with MI results in abnormal heart rhythms and SCD.

### 2.3. Treatment with cLA Increases NOX2 Expression, while Decreasing CX43 Expression in Cardiomyocytes

**Figure 3:**
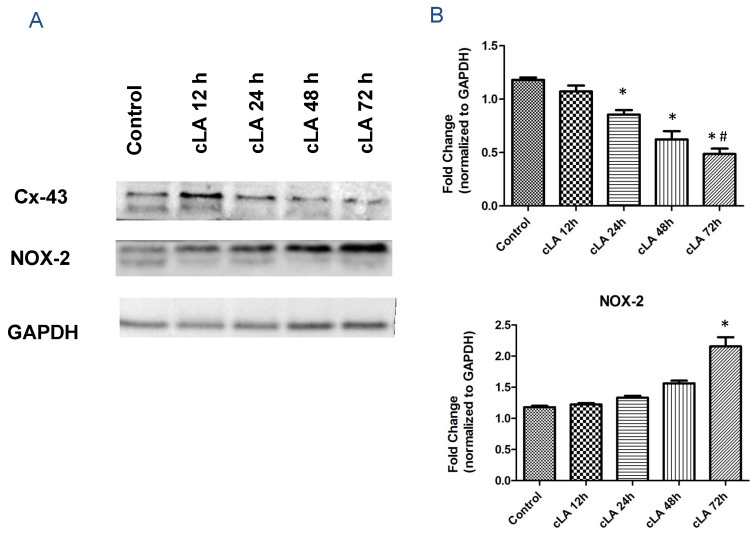
cLA treatment alters Cx-43 level and increases NOX2 level in heart tissue. cLA decreases levels of Cx-43expression in time point treated mice (**A**). Conversely, it increases levels of NOX-expression (**B**). MI surgery in untreated mice (n = 15); MI surgery in cLA-treated mice (n = 35). * *p* < 0.05 vs. control, # *p* < 0.05 vs. cLA 12 h.

**Figure 4:**
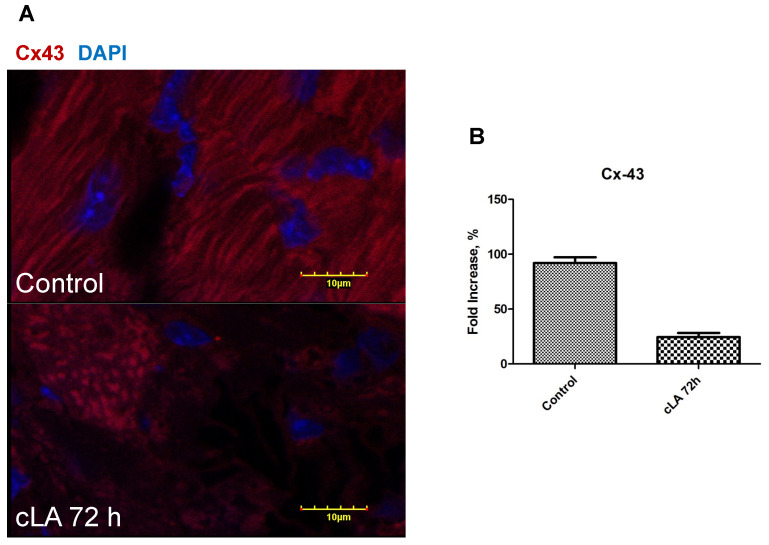
cLA exacerbates connexin-43 disruption in mice after 72 h of treatment. Cx-43 is stained in red, in control (**A**) Cx-43 is represents linear staining, which is disrupted in cLA-treated mice after 72 h of treatment (**A**). Quantitated protein expression reveals that cLA lowers CX-43 expression in mice treated with cLA after 72 h of treatment (**B**). MI surgery in untreated mice (n = 15); MI surgery in cLA-treated mice (n = 35).

Additionally, it is known that ROS plays a role in ventricular arrhythmogenesis by altering Cx43 between myocytes [21,27,28]. Cx43 decreased in a time-dependent manner within the left ventricle in cLA-treated mice (Figure 3A). As early as 24 h following treatment with cLA, Cx43 expression was significantly decreased compared to the control (Figure 3B). In contrast, NOX2 expression significantly increased following treatment with cLA (Figure 3A,B). Further, IHC linear staining of Cx43 was decreased 72 h following cLA treatment (Figure 4A,B). cLA-induced anti-tumorigenic effects have been linked to ROS generation and subsequent apoptosis in animals and cancer cell lines [29,30]. cLA isomers effectively inhibited the growth of cancer cells and this effect was associated with disruption of intercellular Cx43 [31]. Our results further demonstrate that cLA induces ROS within the myocardium within 72 h following treatment. Further, cLA reduces Cx43 expression, causing decreased survival in mice during MI.

## 3. Discussion

**Figure 5:**
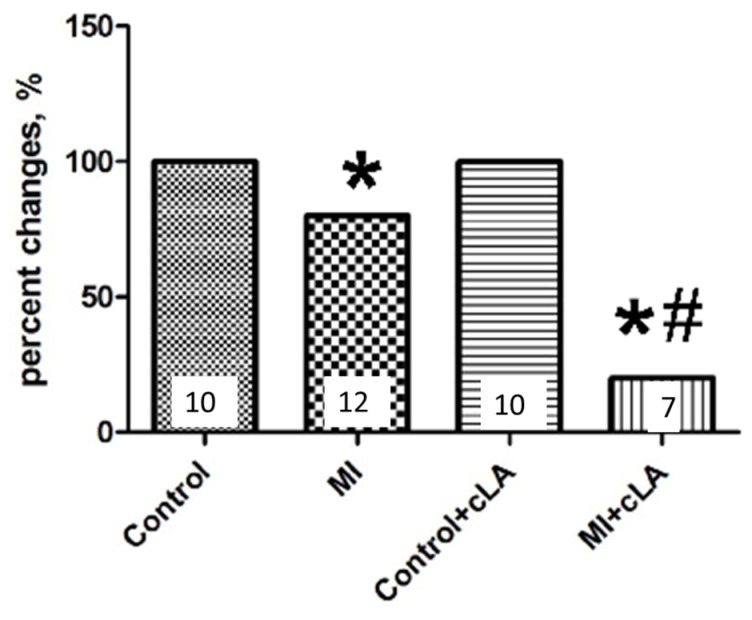
cLA alters survival in mice during myocardial infarction (MI). cLA decreases survival during MI. * *p* < 0.05 vs. control, # *p* < 0.05 vs. MI. MI surgery in untreated mice results in a cumulative survival of 80%, however mice treated with cLA had a cumulative survival of 20% during MI surgery. Numbers on graph represent the number of animals analyzed in each group.

The principal findings of this study are that treatment with cLA disrupts connexin connections between myocytes leading to life-threatening arrhythmias, resulting in VT during MI. The treatment of mice with cLA 3 days prior to MI surgery resulted in a lower survival rate during MI compared to untreated MI mice. This was caused by VT compared to untreated MI mice (Figure 5). SCD can occur without structural heart disease and is attributed in most cases to electrical disturbances leading to abnormal heart rhythms. Cx43 is the most important protein in ventricular gap junctions and reduction in Cx43 results in SCD [32,33,34]. In the heart, myocyte-to-myocyte electrical coupling is mediated via gap junctions, forming the intercellular pathway for proper conductance of electrical excitation in synchronous contraction [16,35,36]. The regulation of Cx43 is under continuous investigation as it is still unclear whether the effects of Ca^2+^ are direct or due to the action of intracellular mediators.

Physiological concentrations of Ca^2+^ are known to regulate the permeability of Cx43 in a calmodulin-dependent manner. Recently it was shown that the lipolysis/lipogenesis balance in white adipocytes is also regulated through Ca^2+^ flux, and that its signaling is controlled through vesicular ATP release [37]. Further, the deuterated form of linoleic polyunsaturated fatty acid (D4-Lnn), a polyunsaturated fatty acid (PUFA), induced cytoprotective effects in an oxygen-glucose deprivation (OGD) ischemic-type model through the activation of the phosphoinositide calcium system [38]. The suppression of damaging proteins and activation of protective genes was also related to an increase in ROS, resulting in an overall inhibition of neuronal apoptosis [38]. Similarly, neuronal cells treated with deuterated PUFAs, completely prevented the ROS-induced effects of oligomeric α-synuclein on lipid peroxidation [39].

**Figure 6:**
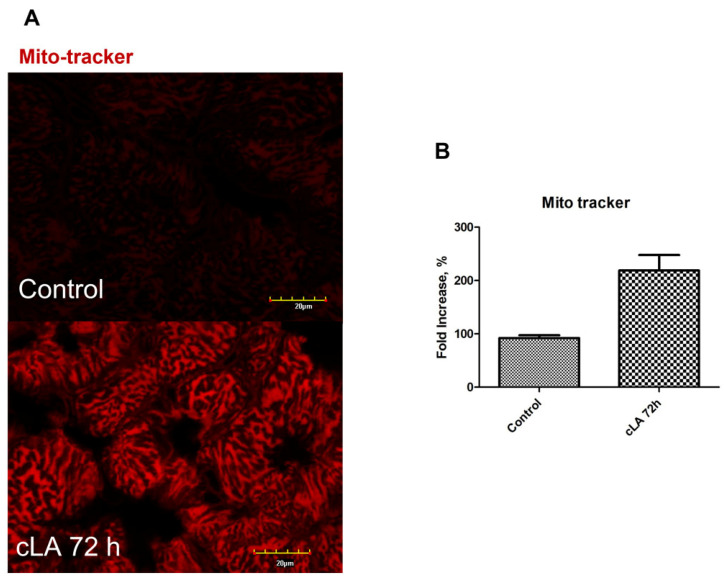
cLA treatment increases ROS generation after 72 h of treatment. Immunohistochemistry images demonstrate MitoTracker staining, which measures ROS generation, which was increased in treated mice with cLA after 72 h of treatment (**A**). Quantitated expression of MitoTracker (**B**).

NOX2 expression in heart tissue has been shown to increase ROS generation and the incidence of arrhythmias [40]. To investigate ROS generation in myocytes, heart slices were stained with MitoTracker. Mice treated with cLA showed ROS generation within 72 h, as demonstrated by a significant increase in MitoTracker staining, compared to control mice (Figure 6A,B). The use of different NOX enzyme isoforms is known to result in different ROS, which highlights the complex role of NOX-derived ROS in in cardiac pathophysiology [41]. However, it has also been reported that NOX2 is a prominent ROS generator and leads to direct myocardial damage, compared to that of other isoforms [42]. Our results demonstrate that increased ROS production at the 72 h time point may be responsible for the disruption of connexins and the development of abnormal heart rhythm following treatment with cLA in MI. Overall, further investigation is warranted as to the consumption of cLA and the potential for increased an risk of VT and SCD with MI.

## 4. Materials and Methods

### 4.1. Animals

The mice were fed standard chow and water ad libitum. All animal procedures were reviewed and approved by the independent Institutional Animal Care and Use Committee (IACUC) of the University of Louisville, School of Medicine (IACUC protocol 14079). In addition, all studies were performed in accordance with the animal care and use guidelines of the National Institutes of Health.

### 4.2. Mouse Model of Myocardial Infarction

Male C57BL/6 mice, when 10–12 weeks old, were anesthetized with isoflurane, intubated, and ventilated with a CWE advanced ventilator (Webster, TX, USA). Body temperature was maintained with an Indus Temperature feedback/surgical table and ECG system. An aseptic procedure was used for the preparation of the surgical site through scrubbing with a 0.8% chlorhexidine solution. A left thoracotomy was performed via the fourth intercostal space and the lungs retracted to expose the heart. After opening the pericardium, the left anterior descending (LAD) coronary artery was ligated with an 8–0 silk suture near its origin between the pulmonary outflow tract and the edge of the atrium. Ligation was deemed successful when the anterior wall of the left ventricle (LV) turned pale. The lungs were inflated by increasing positive end-expiratory pressure, and the thoracotomy side was closed in layers. The lungs were re-expanded, and the chest was closed. The animals were removed from the ventilator and allowed to recover on a heating pad. Mice were checked daily for signs of pain or distress and were given Buprenex at 0.05 mg/kg SQ before the procedure and every 12 h for 48 h. Mice were treated with cLA (10 mg/kg/d-via osmotic mini-pump) 3 days prior to the ligation of the LAD artery.

Mice were treated with cLA (10 mg/kg/d-via osmotic mini-pump) for 12, 24, 48, or 72 h. Following treatment, mice were injected with 50 mg/kg pentobarbital and euthanized. Tissue was harvested for the following experiments.

### 4.3. Western Blot

Heart tissue homogenate (100 μg) was electrophoresed using the SDS-PAGE method as previously described [43,44]. Affinity-purified GAPDH (1:3000) (Trevigen, Gaithersburg, MD, USA), Nox2 (1:1000) (#ab129068-Abcam, Branford, CT, USA), and Cx-43 (1:1000) (#3512S-Cell Signaling, Danvers, MA, USA) antibodies were detected using species-appropriate horseradish peroxidase-labeled secondary antibodies. 

### 4.4. Echocardiography

Transthoracic echocardiography was performed using a VisualSonics ultrasound system (Vevo 2100; VisualSonics, Inc., Toronto, ON, Canada). Left ventricular function was analyzed via the short parasternal axis view. Echocardiograms were performed before MI surgery.

### 4.5. Histology, Immunohistochemistry and Confocal Microscopy

Hearts were collected from mice and thoroughly washed in PBS. Using Peel-A-Way disposable plastic tissue embedding molds (Polysciense Inc, Washington, PA, USA) filled with tissue freezing media (Triangle Biomedical Sciences, Durham, NC, USA), hearts were preserved and stored at −80 °C until analysis. Tissue slices (5 µm in thickness) were made using the Leica CM 1850 Cryocut microtome (Bannockburn, IL, USA). Slices were placed on Superfrost™ Plus glass slides, air-dried, and processed for immunohistochemistry (IHC) staining. Immunohistochemistry and laser-scanning confocal microscopy were used to visualize cLA-induced changes in Cx43 and mitochondrial stress (MitoTracker) expression and localization.

Immunohistochemistry was performed on frozen tissue slices using a standard IHC protocol. Primary antibodies were applied overnight (anti-Cx43). Secondary antibodies labeled with Texas Red (Invitrogen, Carlsbad, CA, USA) were applied for protein immunodetection.

Mitochondrial superoxide generation was assessed by MitoTracker (Red CMXRos) staining, a fluorogenic dye that is taken up by mitochondria. MitoTracker Red CMXRos, tetramethylrhodamine methyl ester, and 10-N-nonyl acridine orange label mitochondria in a manner dependent on the membrane potential, thus giving an indication of mitochondrial stress. The MitoTracker probe, CMXRos Red, is taken up passively by cells. In the mitochondria, the probe is oxidized by superoxide, resulting in the emission of red fluorescence. Fresh frozen 5 μm LV slices were incubated for 45 min at 37 °C with 100 nM MitoTracker (Red CMXRos).

Stained slides were analyzed for fluorescence using a laser scanning confocal microscope (Olympus FluoView-1000, objective 40X, Melville, NY, USA) set at the appropriate filter settings (Tex-red for Cx-43, Cy-3 for MitoTracker). The total fluorescence (red) intensity in 5 random fields (for each experimental sample) was measured with image analysis software off-line (Image-Pro Plus 7.0, Media Cybernetics; Bethesda, MD, USA). The fluorescence intensity values for each experimental group were averaged and presented as fluorescent intensity units (FIU).

### 4.6. Statistical Analysis

Statistical analyses were performed with GraphPad Prism (version 5.0) Statistical Software, and the significance level was set at *p* < 0.05. Values are presented as mean ± SD. Echocardiography and Western blot measurements were analyzed using one-way analysis of variance (ANOVA), followed by Bonferroni’s multiple comparison post hoc analysis. Cx-43 and MitoTracker immunofluorescence intensity were analyzed using two-tailed *t* test with Welch’s correction for simple two group comparisons.

## 5. Conclusions

Our data demonstrate that cLA causes life-threatening arrhythmias in MI. Sudden arrhythmic death is a result of an abnormal heart rhythm. A significant increase in NOX2 expression following cLA treatment in MI results in ROS generation in cardiac tissue. Further, the disruption of Cx43 between myocytes ultimately causes life-threatening arrhythmias, VT, and death during MI. While cLA is generally regarded as safe, individuals susceptible to adverse cardiovascular events such as MI should use caution regarding cLA consumption.

## Data Availability

The data presented in this study are available on request from the corresponding author.
